# Stigma and Access to Mental Healthcare Among US Veterans

**DOI:** 10.7759/cureus.102713

**Published:** 2026-01-31

**Authors:** Justin B Atkins, Ronnie Joseph

**Affiliations:** 1 Medicine, Kirk Kerkorian School of Medicine, University of Nevada, Las Vegas (UNLV), Las Vegas, USA; 2 Emergency Medicine, Kirk Kerkorian School of Medicine, University of Nevada, Las Vegas (UNLV), Las Vegas, USA

**Keywords:** mental health access, primary care integration, ptsd, stigma, telehealth, veterans

## Abstract

Mental health disorders, including posttraumatic stress disorder (PTSD), depression, and substance use disorders, disproportionately affect US military veterans and contribute to increased morbidity and mortality. Despite the availability of Veterans Affairs (VA) mental health services, treatment engagement remains suboptimal. National survey data indicate that while approximately one quarter of veterans screen positive for a probable mental or substance use disorder, fewer than one-third report current mental healthcare utilization. Treatment gaps are especially concerning among high-risk groups, including veterans endorsing suicidal ideation, among whom only a minority engage in current mental health treatment. Stigma, both internalized and anticipated, along with structural barriers such as rural access limitations, system navigation challenges, and concerns regarding confidentiality, significantly contributes to poor utilization of mental health services. This narrative review synthesizes peer-reviewed literature published between 2014 and 2025 examining cultural, attitudinal, and structural barriers to mental healthcare utilization among US veterans and evaluates evidence-based interventions aimed at improving engagement and reducing stigma. Findings consistently demonstrate that internalized stigma, fear of judgment, and masculine identity norms deter help-seeking behaviors, while geographic isolation, administrative complexity, and perceived lack of confidentiality further exacerbate avoidance. Interventions associated with improved engagement include integrated primary care-mental health models, peer navigator programs, tele-mental health services, and targeted anti-stigma initiatives. Addressing both cultural and structural determinants through a multifaceted approach is essential to improving mental healthcare utilization among veterans. Family medicine physicians, particularly in rural and community-based settings, are uniquely positioned to facilitate early identification, reduce stigma, and promote sustained engagement in mental healthcare.

## Introduction and background

Mental health disorders remain a critical public health concern among US military veterans, contributing to significant functional impairment, elevated risk of suicide, and reduced quality of life [1]. Epidemiological data indicate that approximately 30% of veterans experience symptoms consistent with a diagnosable mental health disorder, a prevalence comparable to that observed in the general US adult population [2,3]. Despite this burden, treatment engagement remains limited. Nationally representative data from the 2019-2020 National Health and Resilience in Veterans Study indicate that only 12% of all US veterans report current use of mental health services; even among veterans screening positive for a probable mental or substance use disorder, fewer than one-third (27%) report receiving care [4]. Gaps in engagement are particularly concerning among high-risk populations, as only 36.1% of veterans endorsing current suicidal ideation report active mental health treatment [5].

This gap in treatment engagement has been attributed to a complex interplay of attitudinal, cultural, and structural barriers that inhibit help-seeking behaviors. Stigma, both internalized and anticipated, has been repeatedly identified as a primary barrier to care, with veterans expressing fears of being perceived as weak, concerns regarding confidentiality, and apprehension about potential occupational or institutional consequences associated with mental health disclosure [1,6]. Military culture, particularly among male veterans, emphasizes resilience and self-reliance, which may discourage recognition of mental health symptoms and contribute to delayed care-seeking [2]. Structural barriers are also prominent, including geographic distance from Veterans Affairs (VA) facilities, particularly in rural areas, difficulty navigating eligibility and scheduling systems, and mistrust of providers or administrative processes [7-9]. Collectively, these factors contribute to reduced utilization of mental health services, delays in care delivery even when services are sought, and poorer clinical outcomes, including increased suicide risk.

The purpose of this narrative review is to provide a comprehensive synthesis of current literature on barriers to mental healthcare access among US veterans, categorize these barriers into thematic domains, and evaluate evidence-based strategies that may mitigate these challenges. By identifying key factors affecting care utilization, this review aims to inform clinical practice in family medicine and support the development of targeted interventions to improve mental health outcomes for veterans.

Portions of this review were presented as a poster at the 2026 Nevada Academy of Family Physicians (NAFP) Winter Continuing Medical Education (CME) Meeting in Lake Tahoe, Nevada.

## Review

Attitudinal and cultural barriers

Attitudinal and cultural factors represent some of the most significant barriers to mental health treatment among US veterans. Stigma, both internalized and anticipated, has consistently been identified as a primary deterrent to care-seeking, with veterans expressing fears of being perceived as weak, concerns regarding confidentiality, and apprehension about negative repercussions for career advancement or interpersonal relationships [1,4]. Internalized stigma occurs when veterans adopt societal or cultural negative beliefs about mental illness, leading to self-judgment and shame, whereas anticipated stigma involves expectations of rejection, discrimination, or judgment from others if mental health needs are disclosed [1]. Quantitative studies support these associations; prior research has demonstrated that male veterans with stronger adherence to traditional masculine norms are significantly less likely to engage in mental health treatment, including posttraumatic stress disorder (PTSD)-related care [2].

Military culture is often characterized by values such as resilience, stoicism, loyalty, and self-sacrifice, although these norms are not uniform and may vary across service branches, occupational roles, and unit contexts. While these traits are adaptive during military service, they may inhibit post-service help-seeking behavior [2]. Veterans may perceive psychological symptoms as personal failings, leading to denial, minimization, or avoidance of treatment [8-10]. Help-seeking is often viewed as incompatible with the military ethos of self-reliance, particularly within combat-oriented roles where norms of emotional control and resilience may be more strongly reinforced. This dynamic is especially evident among male veterans, who demonstrate lower rates of mental health service utilization and greater endorsement of masculine norms that discourage expressions of vulnerability [2]. Silvestrini and Chen found that male veterans with higher adherence to traditional masculine ideology were significantly less likely to engage in PTSD treatment, citing concerns over loss of identity and social standing within military and civilian communities [2]. Emerging evidence further suggests that stigma related to mental healthcare is not uniform across diagnoses or treatment types; conditions such as PTSD, which are often framed as responses to external trauma, may be perceived as more acceptable than other mental health diagnoses, whereas conditions associated with emotional vulnerability or internal distress may carry greater stigma, influencing veterans' willingness to seek care and engage in treatment.

Anticipated stigma is especially pronounced among veterans who served in leadership roles or those currently in reserve or guard components, due to concerns that disclosing mental health symptoms may be perceived as compromising security clearance, promotion potential, or unit cohesion [4]. Regardless of whether such outcomes are consistently realized, perceived institutional consequences play a significant role in discouraging disclosure and delaying treatment-seeking. Studies also indicate that stigma contributes to delayed entry into care, resulting in greater symptom severity at the time treatment is initiated [3,9].

Avoidance behaviors associated with trauma-related conditions can further exacerbate attitudinal barriers. Veterans with PTSD may avoid clinical settings that trigger reminders of military experiences, including VA facilities perceived as closely tied to military systems, thereby perpetuating disengagement from care [7].

In addition to stigma, perseverance-oriented traits such as self-reliance or "grit" may independently reduce help-seeking. National survey data indicate that among veterans with high levels of mental dysfunction, those with higher grit were substantially less likely to utilize mental health services than those with lower grit (23% vs. 53%), suggesting that resilience-related traits may delay care even when symptom burden is significant [9]. While these characteristics can confer protective benefits, such as enhanced coping capacity, sustained functioning, and reliance on informal peer supports, they may also impede recognition of the need for professional intervention when distress exceeds individual coping resources.

These attitudinal and cultural barriers remain critical targets for intervention, as they are strongly associated with decreased likelihood of accessing care, reduced adherence to treatment plans, and premature dropout from services. A summary of key attitudinal and cultural determinants of mental healthcare utilization among US veterans is presented in Table 1.

**Table 1:** Attitudinal and cultural determinants of mental healthcare utilization among US veterans This table is an original creation by the authors, synthesizing findings from the cited literature.

Barrier type	Description	Impact on help-seeking	Supporting evidence
Internalized stigma	Veterans endorse negative beliefs about mental illness, viewing it as a weakness	Leads to self-blame, shame, and avoidance of treatment	[1,4]
Anticipated stigma	Fear of negative judgment by peers, leadership, or healthcare providers	Suppresses disclosure of symptoms	[3,4]
Masculinity ideology	Traditional male gender norms emphasizing toughness and emotional control	Reduces willingness to seek therapy, especially among male veterans	[2,10]
Self-reliance/grit	Belief in managing problems independently without external help	Associated with delayed treatment engagement	[9]

Structural and logistical barriers

In addition to cultural and attitudinal determinants, structural and logistical barriers significantly impact mental health service utilization among veterans. These barriers frequently intersect with geographic, administrative, and practical limitations, disproportionately affecting veterans residing in rural areas, those with complex health needs, and individuals unfamiliar with VA navigation processes.

Geographic Barriers and Rural Access

Veterans living in rural regions face increased travel burden due to the limited availability of nearby VA facilities, resulting in longer travel times, transportation costs, and difficulties attending ongoing appointments [5,6]. Approximately one quarter of US veterans reside in rural or highly rural areas, making geographic barriers a population-level concern rather than a niche issue. Cheney et al. reported that rural veterans were more likely to delay or forgo mental health treatment due to distance, limited appointment availability, and reduced access to specialty services compared to urban veterans [6]. National survey data further suggest that rural residence is associated with lower overall mental health service utilization, even after accounting for symptom severity [9]. The limited availability of mental health providers outside major urban centers contributes to unmet need and exacerbates disparities in care engagement [5].

System Navigation Challenges

Complexity in navigating the VA healthcare system is frequently cited as a major barrier to accessing care. Veterans describe difficulty understanding eligibility requirements, scheduling appointments, and coordinating referrals between primary care and specialty mental health services [6,7]. Quantitative studies indicate that a substantial proportion of veterans referred for specialty PTSD care either delay initiation or fail to engage altogether, highlighting the impact of procedural friction on treatment uptake [7]. Navigational burden can be particularly challenging for veterans with cognitive impairments, PTSD, or comorbid physical health conditions, for whom administrative tasks may be overwhelming. Perceptions of inefficiency or lack of personalization within the system have been associated with decreased trust and lower willingness to seek care [8].

Financial and Time Burdens

Although VA mental healthcare is subsidized, indirect costs such as lost wages from taking time off work, transportation expenses, and childcare responsibilities can deter veterans from seeking regular treatment [6]. Veterans who are employed or transitioning from active duty to civilian occupations often experience competing demands, such as inflexible work schedules, caregiving responsibilities, educational or vocational training commitments, and administrative requirements associated with employment or benefits, that limit availability to attend mental health appointments, particularly when recurring weekly sessions are required.

Continuity and Wait Times

Structural concerns related to continuity of care, including long wait times for appointments and frequent changes in treating clinicians, have been shown to decrease engagement. Veterans frequently report frustration with having to repeatedly recount personal histories to new providers, a process that can be emotionally taxing and reinforce avoidance behaviors [7,8]. Disruptions in continuity are consistently associated with lower trust, reduced therapeutic alliance, and delayed treatment initiation among veteran populations. Long wait times may further reduce motivation to engage in care and contribute to symptom progression prior to treatment initiation [11].

Digital Infrastructure Limitations

While tele-mental health services have expanded access, particularly for rural veterans, they remain underutilized by some populations due to limited broadband availability, lack of familiarity with digital platforms, or preference for in-person care [6]. National data indicate that disparities in digital access persist, with older veterans and those living in rural or underserved regions less likely to have reliable high-speed internet. Although evidence-based trauma-focused treatments have been successfully adapted for video-based delivery, barriers related to digital literacy, technology reliability, and privacy concerns continue to limit the reach and effectiveness of tele-mental health services for certain veterans.

Collectively, these logistical and structural obstacles compound existing attitudinal barriers, creating a multifaceted web that impedes engagement with mental health services. Effective interventions must therefore address the practical realities of access, navigation, and continuity, rather than focusing solely on individual beliefs or motivation. A summary of key structural and logistical barriers influencing mental healthcare access among US veterans is presented in Table 2.

**Table 2:** Key structural and logistical barriers influencing mental healthcare access among US veterans This table is an original creation by the authors, synthesizing findings from the cited literature.

Barrier	Mechanism	Effect on access	Supporting studies
Rural residence	Long travel times; limited clinics	Reduced appointment adherence, increased dropout	[6]
Digital access limitations	Lack of broadband for telehealth	Limits remote therapy utilization	[6]
Navigation complexity	Difficulty understanding Veterans Affairs processes	Discourages initiation of care	[7]
Provider turnover	Lack of continuity	Distrust, frustration, disengagement	[8]

Institutional and systemic barriers

Institutional and systemic barriers within the VA healthcare system and broader governmental structures contribute significantly to reduced mental health treatment utilization among veterans. These barriers often stem from institutional processes that inadvertently deter engagement, including concerns about confidentiality, mistrust of providers, administrative burden, and negative perceptions of the disability compensation system.

Mistrust of the VA System and Providers

Mistrust of institutional healthcare systems has been identified as a major deterrent to care-seeking, particularly among veterans with prior negative experiences or unmet expectations [6]. Cheney et al. found that veterans frequently perceive the VA as impersonal and bureaucratic, citing limited provider continuity and insufficient attention to individual needs as barriers to engagement [6]. While similar concerns are observed in civilian healthcare systems, mistrust within the VA context may carry distinct perceived consequences, including fears of being "labeled" in the medical record in ways that could affect service-connected benefits, employment opportunities, or future eligibility determinations [4]. These perceptions may disproportionately influence veterans who rely on the VA for both healthcare and financial or occupational support.

Confidentiality Concerns

Apprehension regarding confidentiality is a recurring theme across the literature. Veterans commonly express concerns that mental health diagnoses may be documented in ways that could affect career advancement, security clearance status, or eligibility for firearms [4,7]. Such concerns are particularly salient among veterans who served in leadership or combat roles or who continue to serve in the National Guard or Reserves [4]. Although the actual consequences of receiving mental health treatment, such as evidence-based therapies for PTSD, vary widely depending on role, jurisdiction, and employer policy, concerns about how mental health information may be interpreted or used are often poorly understood and can meaningfully deter disclosure and care-seeking. Importantly, perceived risk alone has been shown to influence behavior, regardless of whether adverse outcomes are consistently realized.

Perceived Consequences of the Disability Compensation Process

The VA disability claims process itself may function as a barrier to mental healthcare engagement. Hooyer describes the concept of the "trauma pitch", in which veterans are required to repeatedly recount traumatic experiences during compensation evaluations, a process frequently described as retraumatizing [8]. Veterans report that this requirement may discourage treatment initiation or continuation to avoid psychological distress. Additionally, some veterans perceive a misalignment between the VA's dual role as both treatment provider and benefits evaluator, which can undermine trust in the therapeutic relationship and reduce willingness to engage in care [8].

Administrative and Procedural Burdens

Administrative complexity, including appointment scheduling, referral requirements, documentation demands, and care coordination, poses a significant barrier to sustained engagement [6]. These burdens may be especially challenging for veterans with PTSD, depression, or cognitive impairment, who may experience symptoms such as avoidance, impaired concentration, or emotional dysregulation that complicate navigation of bureaucratic systems [7]. Quantitative evidence suggests that administrative burden and care fragmentation are associated with lower treatment adherence and increased likelihood of delayed initiation or premature dropout from mental health services [11].

Impact on Treatment Engagement

Collectively, institutional barriers affect not only initial access but also engagement across the treatment continuum. Veterans report frustration with procedural inefficiencies and provider turnover, which can erode therapeutic alliance and reduce the perceived value of care [6,7]. National survey data indicate that even among veterans who meet criteria for common mental health conditions, a substantial proportion discontinue care or fail to engage consistently following referral [9,11]. When institutional processes are experienced as invalidating, stigmatizing, or overly burdensome, veterans are more likely to disengage prematurely or avoid re-entry into care following symptom exacerbation [8]. A summary of key institutional and systemic barriers influencing mental healthcare access among US veterans is presented in Table 3.

**Table 3:** Key institutional and systemic barriers influencing mental healthcare access among US veterans This table is an original creation by the authors, synthesizing findings from the cited literature.

Systemic factor	Description	Impact	Evidence
Disability claims trauma narrative requirement	Veterans must relive trauma repeatedly to justify benefits	Re-traumatization, avoidance of Veterans Affairs services	[8]
Confidentiality concerns	Belief that disclosures affect employment or firearms rights	Avoidance of psychiatric treatment	[4]
Administrative burden	Complex referral and scheduling process	Lower treatment adherence	[6]

**Figure 1:**
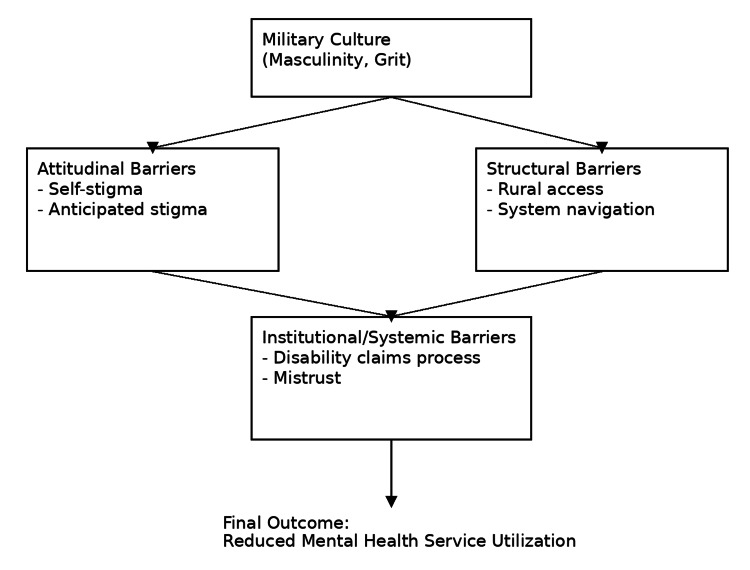
Conceptual framework of attitudinal, structural, and institutional barriers to mental healthcare access among US veterans Conceptual framework illustrating attitudinal, structural, and institutional barriers contributing to reduced mental health service utilization among US veterans. Military cultural factors influence both attitudinal and structural barriers, which interact with institutional and systemic processes to impede care engagement. This figure is an original creation by the authors.

Institutional and systemic factors thus represent a critical dimension of the barriers to mental healthcare among veterans, requiring organizational and policy-level interventions to restore trust and facilitate seamless access to services. A conceptual framework of barriers to mental healthcare for veterans is outlined in Figure 1.

Disparities among high-risk subpopulations

While attitudinal, structural, and institutional barriers to mental healthcare affect the veteran population broadly, their impact is not uniform. Certain subpopulations, including female veterans, rural veterans, aging veterans, and racial and ethnic minority groups, experience unique barriers that contribute to disparities in treatment engagement and outcomes. Emerging literature also suggests that experiences may vary by type of service, occupational role, and neurocognitive status, including the presence of traumatic brain injury or other neurodiversity-related conditions, which can further complicate navigation, communication, and engagement in care. However, data across these dimensions remain heterogeneous, and a comprehensive examination of service-specific or neurodiversity-related disparities was beyond the scope of this review, representing an important area for future research.

Female Veterans

Female veterans represent one of the fastest-growing segments of the veteran population, yet they are less likely than their male counterparts to seek mental health treatment due to concerns regarding stigma and gender-specific experiences of military service [10]. Although some studies suggest female veterans are more likely to acknowledge mental health symptoms than males, they report greater discomfort with VA environments perceived as male-dominated and express concerns regarding privacy and safety when discussing sensitive experiences such as military sexual trauma [6]. Additionally, anticipated stigma related to gendered expectations within military culture may lead to underreporting of symptoms and delayed care-seeking [4]. Tailored interventions that provide gender-sensitive care and reduce environmental barriers may improve engagement among female veterans.

Rural Veterans

Veterans residing in rural areas, commonly defined using US federal classifications as individuals living outside metropolitan or micropolitan regions, face compounded structural and cultural barriers to care, including geographic isolation, limited provider availability, and lower trust in government institutions [6]. Approximately one quarter of US veterans reside in rural or highly rural areas, making these barriers particularly salient at the population level. Rural veterans often endorse stronger beliefs in self-reliance and stoicism, which may intensify internalized stigma and reduce treatment-seeking behavior [2,9]. Disparities in broadband access further limit the utilization of tele-mental health services, exacerbating existing gaps in care [6].

Aging Veterans

Older veterans may face distinct barriers including digital literacy limitations, reduced familiarity with mental health terminology, and attribution of symptoms to aging rather than psychiatric disorders [11]. They may also be less likely to identify emotional distress as a treatable condition. Furthermore, older veterans may encounter mobility-related challenges that impede travel to VA facilities [5].

Racial and Ethnic Minority Veterans

Minority veterans often experience additional barriers to care stemming from systemic inequities, cultural mistrust of healthcare institutions, and perceptions of discrimination [3]. Studies indicate that minority veterans are less likely to receive guideline-concordant care and more likely to discontinue treatment prematurely compared to White veterans [3]. Additionally, lack of culturally competent care and perceived racial bias within institutional settings may contribute to disengagement.

Veterans With High Perseverance Traits

While perseverance and resilience are considered adaptive traits, veterans with high self-reliance or "grit" scores may be less likely to seek professional care, preferring to manage symptoms independently [9]. This avoidance may persist even in the presence of clinically significant impairment. At the same time, these traits can confer important protective benefits, including enhanced coping capacity, sustained occupational functioning, and reliance on informal supports such as peer networks or mission-oriented activities. However, when distress exceeds individual coping resources, strong norms of self-reliance may delay recognition of need or engagement with formal treatment. The presence of these disparities underscores the necessity of targeted, population-specific approaches that acknowledge and leverage existing strengths while reducing barriers to timely mental healthcare engagement among veterans. A "one-size-fits-all" intervention is unlikely to adequately address the nuanced needs of diverse veteran subgroups.

Evidence-based interventions and emerging models of care

Efforts to increase mental health treatment engagement among US veterans have increasingly focused on interventions that target both attitudinal and structural barriers. The literature supports a multifaceted approach integrating clinical, organizational, and policy strategies designed to reduce stigma, improve access, and enhance continuity of care. 

Primary Care-Mental Health Integration

Co-locating behavioral health providers within primary care settings has been shown to reduce stigma by normalizing mental health treatment as part of routine medical care [5]. Integrated care models facilitate warm handoffs between providers, reduce referral burden, and have been associated with increased initiation and retention in treatment [11]. This approach is particularly effective in family medicine settings, where continuous patient-provider relationships facilitate trust and the early identification of psychosocial needs.

Peer Support Programs

Peer navigator models, in which trained veterans provide outreach, support, and mentorship to other veterans seeking care, have demonstrated improvements in treatment engagement and satisfaction [6]. Peer providers are often viewed as more credible and relatable due to shared military experiences, which can reduce anticipated stigma and mistrust of institutional systems [1]. Peer involvement has also been shown to reduce dropout rates among veterans with PTSD. Both government-supported peer programs and community- or charity-based peer initiatives appear to confer benefits, though they may differ in structure and perceived proximity to formal institutions. Some veterans may view community-based or veteran-led peer groups as less bureaucratic or lower risk, potentially enhancing trust and sustained engagement. However, comparative data directly evaluating outcomes between government-provided and non-governmental peer support models remain limited, and further research is needed to clarify differential effects on stigma reduction and retention.

Tele-Mental Health Expansion

Telehealth services have emerged as one of the most impactful strategies for improving access to care, particularly for rural veterans [6]. Studies indicate that tele-mental health reduces geographic and scheduling barriers and has demonstrated clinical effectiveness comparable to in-person treatment [11]. From a patient perspective, telehealth may reduce indirect costs, such as travel time, transportation expenses, and lost wages, while increasing convenience and flexibility. For healthcare systems and providers, tele-mental health can improve scheduling efficiency and expand reach, though it may also introduce challenges related to technology infrastructure, reimbursement models, clinician workload, and the suitability of remote care for certain clinical presentations. Additionally, while remote care options may mitigate anticipated stigma by offering greater privacy, they do not fully eliminate access disparities related to digital literacy or broadband availability.

Anti-stigma Interventions

Targeted anti-stigma campaigns, especially those framed within the context of military values such as strength and readiness, have demonstrated effectiveness in increasing willingness to seek care [4]. Interventions that include testimonials from respected military peers or leaders who have successfully used mental health services are particularly impactful in changing attitudes [2].

Streamlined Administrative Processes

Efforts to simplify the VA disability claims process and reduce redundant trauma recounting are being implemented to address systemic barriers identified in qualitative reports [8]. Policy interventions that reduce administrative burden and emphasize trauma-informed care have been associated with improved patient satisfaction and treatment adherence.

Routine Screening and Proactive Outreach

Routine mental health screening during primary care visits increases the detection of psychological distress and facilitates early intervention [5]. Furthermore, proactive outreach from VA care coordinators, particularly following transitions from active duty or hospitalization, has been associated with reduced suicide risk and greater engagement in outpatient mental health services [3].

Culturally Tailored Care Models

Recognizing disparities among veteran subpopulations, emerging models emphasize culturally competent care, gender-specific treatment options, and services that address the unique needs of minority and female veterans [6]. Tailoring services to individual identities and experiences has been shown to increase engagement and improve clinical outcomes [10].

Summary

Collectively, these interventions underscore the necessity of a comprehensive approach that addresses both psychological and logistical barriers. Evidence supports the integration of mental health into primary care settings, expansion of telehealth infrastructure, implementation of peer support programs, and the development of stigma-reduction initiatives tailored to military culture as effective strategies for enhancing treatment engagement. A summary of key evidence-based interventions and emerging models of care is depicted in Table 4.

**Table 4:** Evidence-based interventions to improve mental healthcare engagement among US veterans This table is an original creation by the authors, synthesizing findings from the cited literature.

Intervention	Target barrier	Evidence of effectiveness	Reference
Primary care-mental health integration	Reduces stigma, improves access through co-location	Increased treatment initiation and retention	[5]
Peer support navigators	Reduces anticipated stigma, improves trust	Improved engagement and satisfaction	[6]
Tele-mental health	Addresses geographical barriers	Equivalent outcomes to in-person care	[7]
Anti-stigma campaigns	Reduces cultural and internalized stigma	Improved attitudes toward treatment	[2]

## Conclusions

Mental health disorders remain highly prevalent among US military veterans and contribute to significant morbidity, impaired functioning, and elevated suicide risk. Despite the availability of services within the Veterans Health Administration, utilization of mental health treatment remains suboptimal due to the combined influences of stigma, cultural identity, structural barriers, and institutional factors. This review demonstrates that barriers to care are multifactorial and interdependent, requiring comprehensive and integrated solutions.

Evidence suggests that interventions targeting both attitudinal and practical barriers yield the greatest improvements in treatment engagement. Integrated primary care-mental health models, tele-mental health services, and peer support programs have been shown to reduce stigma, improve continuity of care, and enhance accessibility, particularly for rural and underserved populations. Anti-stigma interventions that frame help-seeking within the context of strength, resilience, and readiness are effective in shifting cultural attitudes within the veteran community.

Family medicine physicians are uniquely positioned to play a central role in addressing these gaps due to their longitudinal relationships with patients, scope of practice across physical and mental health domains, and presence in both VA and community settings. By incorporating routine mental health screening, collaborative care models, and trauma-informed communication strategies, family medicine can serve as a gateway to early identification, engagement, and retention in care.

Future directions should focus on scaling tele-mental health infrastructure across the veteran healthcare system while prioritizing rural and underserved populations where access barriers are greatest. Expansion of peer-led initiatives and development of culturally tailored care models are also needed to address the diverse needs of female, minority, rural, and other historically underserved veteran groups. At the same time, tele-mental health should be viewed as a complementary modality rather than a universal solution, as limitations related to digital access, clinical appropriateness, patient preference, and continuity of care persist. Continued research is needed to evaluate the long-term impact of these interventions and to identify strategies that personalize care while reducing systemic barriers. A multifaceted approach integrating clinical practice, policy reform, and veteran-centered program design has the potential to significantly improve mental health outcomes and reduce disparities across the veteran population.
